# LncRNA HOXC-AS1 Sponges miR-99a-3p and Upregulates MMP8, Ultimately Promoting Gastric Cancer

**DOI:** 10.3390/cancers14143534

**Published:** 2022-07-20

**Authors:** Yue Jiang, Xiangpan Li, Yu Yang, Jiajun Luo, Xunshan Ren, Jingwen Yuan, Qiang Tong

**Affiliations:** 1 Department of Gastrointestinal Surgery I Section, Renmin Hospital of Wuhan University, Wuhan 430060, China; rosyjy@whu.edu.cn (Y.J.); yy711@whu.edu.cn (Y.Y.); jiajunluo@whu.edu.cn (J.L.); yuanjingwen718@whu.edu.cn (J.Y.); 2 Department of Oncology, Renmin Hospital of Wuhan University, Wuhan 430060, China; lixiangpan@whu.edu.cn; 3 Department of Orthopedics, Renmin Hospital of Wuhan University, Wuhan 430060, China; 2016302180209@whu.edu.cn

**Keywords:** gastric cancer, HOXC-AS1, miR-99a-3p, matrix metallopeptidase 8, ceRNA network

## Abstract

**Simple Summary:**

Long noncoding RNAs, including the HOXC Cluster Antisense RNA 1 (HOXC-AS1), are reported to be critical during the occurrence and progression of gastric cancer. We examined cells and tissues for the expression of HOXC-AS1 and correlated the expression levels with the disease specific survival of the gastric cancer patients. We also identified the interaction between HOXC-AS1 and miR-99a-3p, as well as matrix metalloproteinase 8 (MMP8) by dual-luciferase reporter gene assays. Western blot and qRT-PCR were conducted to verify the alteration in expression levels, while Cell Counting Kit-8 assay and colony formation assay were performed to explore the influences on gastric cancer cells. Overexpression of HOXC-AS1 would accordingly sponge greater quantities of miR-99a-3p, leading to the upregulation of MMP8, eventually facilitating the progress of gastric cancer.

**Abstract:**

Gastric cancer (GC) is among the most lethal tumors worldwide. Long noncoding RNAs (lncRNAs) are reported to be critical during the occurrence and progression of malignancies. The HOXC cluster antisense RNA 1 (HOXC-AS1) has been suggested to participate in the genesis and development of GC. Therefore, we examined GC cells and tissues for the expression of HOXC-AS1 and correlated the expression levels with the disease specific survival of the patients, finding that HOXC-AS1 was overexpressed and probably had a tendency of leading to a poor prognosis. The Cell Counting Kit-8 assay and colony formation assay were then performed under knockdown of HOXC-AS1, revealing that cell proliferation of GC was distinctly decreased. Afterwards, miR-99a-3p was predicted to bind with HOXC-AS1 by DIANA tools. We carried out dual-luciferase reporter gene assays to identify the interaction between them. After knockdown of HOXC-AS1, miR-99a-3p was clearly overexpressed in GC cells. In addition, matrix metalloproteinase 8 (MMP8) was shown to be combined with miR-99a-3p using TargetScan. Similar experiments, along with western blot, were conducted to validate the correlation between miR-99a-3p and MMP8. Finally, rescue experiments for CCK-8 were completed, disclosing that HOXC-AS1 promoted cell progression of GC through sponging miR-99a-3p followed by subsequent upregulation of MMP8.

## 1. Introduction

Gastric cancer (GC) is ranked fifth in incidence and fourth in mortality among numerous malignant tumors around the world [1]. Surgery, radiotherapy, chemotherapy and biologic therapy have all been utilized clinically in the treatment of GC [2]. Despite successes achieved in the therapy of GC, recurrences are fairly common, leading to a poor prognosis [3]. Accordingly, clarifying the etiology of GC and recognizing updated targets for developing new treatment methods are necessary.

Long noncoding RNAs (lncRNAs) have more than 200 nucleotides without evidence of encoding peptides [4,5]. However, they are involved in regulating the translation of genes that code proteins [5]. Recently, lncRNAs have been implicated to take part in the occurrence and development of various cancers, including GC [6,7]. LncRNAs have been reported to play an essential part in apoptosis, angiogenesis and epithelial mesenchymal transition (EMT) in GC [8]. LncRNA MIR210HG was found to associate with DExH-box helicase 9 (DHX9) and enhance the occupancy of the DHX9/c-Jun complex on the promoter of matrix metallopeptidases (MMPs) resulting in the promotion of invasion and migration of GC cells [9]. Moreover, Hypoxia Inducible Factor-1α (HIF-1α) fostered LINC01355 expression through binding its promoter region, which is overexpressed in GC cells and tissues, likely manifesting a worse prognosis [10]. In addition, silencing CRNDE in M2-polarized macrophage-derived exosomes (M2-exo) enhanced the sensitivity of GC cells to cisplatin [11]. Meanwhile, upregulation of lncFEZF1-AS1 led to the promotion of proliferation and autophagy in GC cells, while downregulation restrained cancer cell proliferation and enhanced sensitivity to 5-FU [12]. LncRNA SLCO4A1-AS1 post-transcriptionally regulated the X-linked inhibitor of apoptosis (XIAP) by functioning as a competing endogenous RNA (ceRNA) in GC that sponges miR-149 [13]. Autophagy inhibition resulting from increased MALAT1 affected intercellular interactions between GC cells and fibroblasts, facilitating proliferation of tumors [14].

LncRNA HOXC cluster antisense RNA 1 (HOXC-AS1) was reported to sponge miR-4651 and increase expression of FOXO6, promoting advancement of nasopharyngeal carcinoma [15]. Knockdown of HOXC-AS1 restrained proliferation of castration-resistant prostate cancer through repression of androgen receptors and their diverse expression [16]. Moreover, it is worth noting that the expression of HOXC-AS1 was attributed to the suppression of EIF4AIII, which was shown to facilitate cell proliferation and EMT, while blocking apoptosis in GC [17]. Knockdown of HOXC-AS1 inhibited GC cell growth and metastasis, eventually leading to negative regulation of MYC expression [18]. However, the character of HOXC-AS1 in the ceRNA network remains indistinct. MiR-99a-3p was reported to be upregulated by diaporine A, suppressing proliferation of non-small cell lung cancer (NSCLC) cells [19]. Matrix metalloproteinase 8 (MMP8) was considered as a tumor promoter in various tumors. In this study, we further verified the expression and clarified the effects of HOXC-AS1 in GC, successfully establishing a new ceRNA network in the regulation of GC.

## 2. Materials and Methods

### 2.1. Database and Online Tools

Expression of HOXC-AS1, miR-99a-3p and MMP8 in normal and tumorous gastric tissues were found in the Cancer Genome Atlas (TCGA). The possible binding site of HOXC-AS1 and miR-99a-3p was forecasted by LncBase Predicted v.2 of DIANA Tools (http://carolina.imis.athena-innovation.gr/diana_tools/web/index.php, accessed on 14 October 2021). The possible combining site of miR-99a-3p and MMP8 was forecast by TargetScan (http://www.targetscan.org/vert_80/ accessed on 14 October 2021).

### 2.2. Cell Culture

AGS, SGC7901, SNU1 and MKN45 cell lines were bought from American Type Culture Collection (ATCC, Manassas, VA, USA). The AGS cell line was cultured in Dulbecco’s Modified Eagle Medium (DMEM) /F-12 (ref: G4610, Servicebio, Wuhan, China) while the SGC7901, SNU1 and MKN45 cell lines were cultured in RPMI1640 (ref: G4530, Servicebio, Wuhan, China), along with 10% fetal bovine serum (FBS, ref: 10099141C, ThermoFisher, Waltham, MA, USA) and 1% antibiotics (Penicillin and Streptomycin) (ref: G4003, Servicebio, Wuhan, China). Cells were incubated at 37 °C with 5% CO_2_.

### 2.3. Cell Transfection

The siRNA vectors against HOXC-AS1 (siHOXC-AS1-1, ref: siG150728015810, and siHOXC-AS1-2, ref: siG150728015828) were used to knock down HOXC-AS1. Scrambled siRNA (siNC, ref: siN0000001) served as a negative control. MiR-99a-3p mimics (ref: miR10004511) and inhibitors (ref: miR20004511) were employed to upregulate the expression level of miR-99a-3p. All the materials mentioned above were synthesized by RiboBio (Guangzhou, China). For upregulation of MMP8, we inserted the full length of MMP8 into pcDNA3.1 vectors (ref: V79520, Invitrogen, Waltham, MA, USA). Empty plasmids were considered as a negative control. Before transfection, cells were seeded in 6-well plates without antibiotics for 24 h. Transfections of microRNA mimics and inhibitors, siRNAs and plasmids were all performed with Lipofectamine 2000 (ref: 11668019, ThermoFisher, Waltham, MA, USA) as the manufacturer’s instruction required.

### 2.4. Quantitative Real-Time PCR (qRT-PCR)

Total RNA was extracted by TRIzol reagent (ref: 15596026, ThermoFisher, Waltham, MA, USA) following the manufacturer’s instructions. Reverse transcription was performed with the First Strand cDNA Synthesis Kit (ref: G3330, Servicebio, Wuhan, China). PCR was implemented with SYBR Green qPCR Master Mix (ref: G3320, Servicebio, Wuhan, China). Sangon Biotech (Shanghai, China) compounded all the used primers. The 2-∆∆Ct method was conducted to identify the expression levels of related genes.

The sequences of used primers were indicated as below: HOXC-AS1 (forward): 5′-CAACTCCATCTCTGCGACAC-3′, HOXC-AS1 (reverse): 5′-AACAAGCTACTTGCCCACGA-3′; miR-99a-3p (forward): 5′-CGCTTCTATGGGTCTGGTCG-3′, miR-99a-3p (reverse): 5′-GTGTCGTGGAGTCGGCAATT-3′; MMP8 (forward): 5′-GGAAGGCAGGAGAGGTTGTC--3′, MMP8 (reverse): 5′--GTTGAAAGGCATGGGCAAGG-3′; GAPDH (forward): 5′-CACCATTGGCAATGAGCGGTTC-3′, GAPDH (reverse): 5′-AGGTCTTTGCGGATGTCCACGT-3′; GAPDH was utilized as an endogenous control.

### 2.5. Luciferase Reporter Assays

We carried out luciferase reporter assays to validate the interactions among HOXC-AS1, miR-99a-3p and MMP8. HOXC-AS1 was cloned into the pGL3-Basic (ref: E1751) and the pRL-TK (ref: E2241) vector to construct the reporters. Then, miR-99a-3p mimics and the reporters were transfected into AGS and SGC7901 cell lines. Finally, luciferase activity was quantified and analyzed with the Dual-Luciferase Assay System (ref: E1910, Promega, Madison, WI, USA). The combination between MMP8 and miR-99a-3p was performed similarly as mentioned above.

### 2.6. CCK-8 Assay

We inoculated cells after transfection at a density of 2 × 100 cells per well into 96-well plates for the Cell Counting Kit-8(CCK-8) assay. The cells were then cultivated for 0, 24, 48 and 72 h. Every well was supplemented with 10 μL CCK-8 reagent (ref: G4103, Servicebio, Wuhan, China) at different incubation times, then cultured for another hour. Later, absorbance at 450 nm was measured by a standard microplate reader (EnSight, Perkin Elmer, Waltham, MA, USA).

### 2.7. Colony Formation Assay

The AGS or SGC7901 cells were inoculated into 6-well plates at a density of 100 cells per well after transfection. The plates were then cultured for 2 weeks at 37 °C. Culture was terminated when visible colonies appeared in the plates. We next removed the supernatant and rinsed the cells carefully with PBS (ref: G4202, Servicebio, Wuhan, China). Afterwards, we fixed the cells with 4% paraformaldehyde (ref: 8187150100, Sigma, Germany). The fixative solution was removed and a proper amount of GIMSA solution (ref: 10092013, ThermoFisher, Waltham, MA, USA) was added for 15 min. The dye was washed away with running water slowly and the plates were exposed to air to dry. Finally, the amounts of colony formation were calculated.

### 2.8. Western Blot

Cells were washed with PBS and lysed with radioimmunoprecipitation assay RIPA) buffer (ref: G2022, Servicebio, Wuhan, China). Samples involving equivalent total proteins were resolved on 15% SDS polyacrylamide denaturing gel, then transferred to nitrocellulose membranes. Later, the membranes were blocked with 5% skim milk in Tris Buffered saline Tween buffer(TBST) for 1 h, followed by incubation overnight at 4 °C with anti-MMP8 antibody (1:1000) (ref: ab81286, Abcam, Cambridge, UK) and goat anti-rabbit IgG secondary antibody (1:5000) (ref: G1213, Servicebio, Wuhan, China). Finally, the membrane was visualized by using Omni-ECL TMPico Light Chemiluminescence Kit (ref: SQ201, EpiZyme, Shanghai, China).

### 2.9. Statistical Analysis

Graphpad Prism 8 and R version 3.6.3 were used for statistical analysis. All values were presented as means ± standard deviation (SD). The Student’s t-test was employed to assess the statistical differences between two independent groups. Spearman’s correlation coefficient analysis was conducted to access the expression relationship between different RNAs. Kaplan–Meier was utilized to conduct survival analysis and a log-rank test was employed for identifying the statistical differences. Experiments were repeated three times. *p* < 0.05 was supposed statistically significant.

## 3. Results

### 3.1. HOXC-AS1 Was Overexpressed in GC with Poor Prognosis

**Figure 1:**
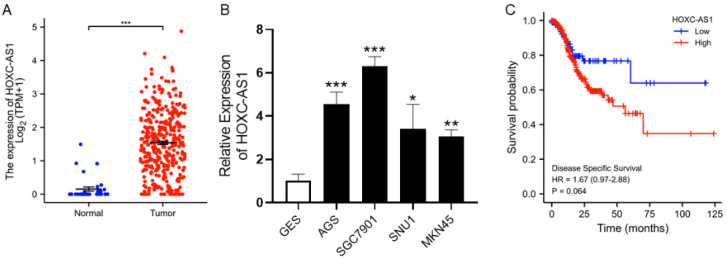
HOXC-AS1 was overexpressed in GC with poor prognosis. (**A**) HOXC-AS1 expression in normal and tumorous gastric tissues from TCGA; (**B**) HOXC-AS1 expression in normal gastric epithelial cells (GES) and GC cell lines (AGS, SGC7901, SNU1 and MKN45) by qRT-PCR; (**C**) Kaplan-Meier analysis for disease specific survival of GC patients based on the expression of HOXC-AS1. * *p* < 0.05; ** *p* < 0.01; *** *p* < 0.001.

The expression of HOXC-AS1 in GC was determined from the TCGA database and it was significantly upregulated in tumorous compared to normal gastric tissues (Figure 1A). We performed qRT-PCR to verify elevated HOXC-AS1 expression in cell lines and discovered that, compared to normal gastric epithelial cells, HOXC-AS1 was overexpressed in several GC cell lines, including AGS, SGC7901, SNU1 and MKN45 (Figure 1B). Not only that, the high expression group also tended to have a shorter disease specific survival (DSS) than the low expression group (Figure 1C). These outcomes forecast that HOXC-AS1 is upregulated in GC, presumably leading to a poor prognosis.

### 3.2. Downregulation of HOXC-AS1 Inhibited GC Cell Proliferation

**Figure 2:**
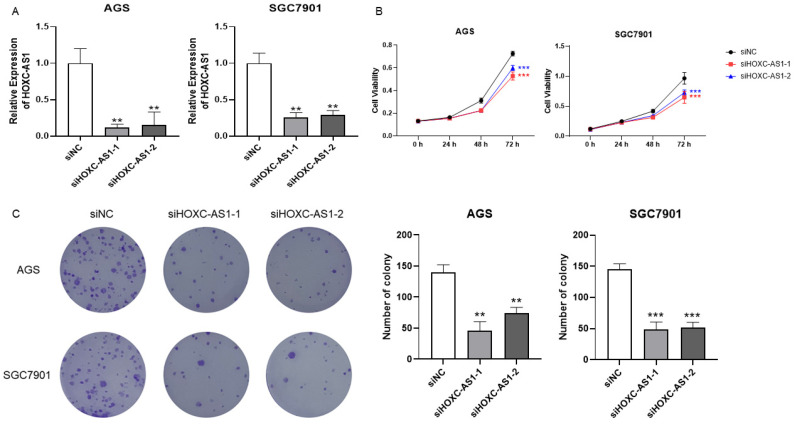
Downregulation of HOXC-AS1 inhibited GC cell proliferation and colony formation. (**A**) Verification of HOXC-AS1 knockdown in AGS and SGC7901 cell lines by qRT-PCR. (**B**) Results of CCK-8 assays after transfection of siRNAs. (**C**) Results of colony formation assays after knockdown of HOXC-AS1. ** *p* < 0.01; *** *p* < 0.001.

To disclose the function of HOXC-AS1 in GC, two siRNAs targeting HOXC-AS1 (siHOXC-AS1-1 and siHOXC-AS1-2) were transfected into AGS and SGC-7901 cell lines to knockdown its expression. It was shown by qRT-PCR that HOXC-AS1 expression in the cell lines was significantly decreased using both siRNAs (Figure 2A). Since high expression of HOXC-AS1 closely associated with shorter DSS in GC, we presumed HOXC-AS1 to participate in the initiation and progression of GC. The CCK-8 assay showed that the optical density (OD) of siHOXC-AS1-transfected AGS and SGC7901 cells lines was distinctly decreased (Figure 2B), which indicated reduced proliferation in GC cells after knockdown of HOXC-AS1. Finally, a colony formation experiment was also performed. In accordance with the previous results, siHOXC-AS1-transfected AGS and SGC7901 cells formed fewer colonies, furtherly proving that downregulation of HOXC-AS1 led to suppression of GC cell proliferation (Figure 2C).

### 3.3. HOXC-AS1 Sponged miR-99a-3p in GC

**Figure 3:**
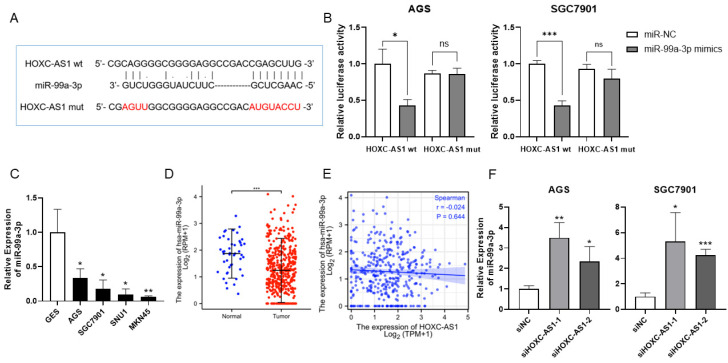
HOXC-AS1 sponged miR-99a-3p in GC. (**A**) The predicted binding site of HOXC-AS1 and miR-99a-3p using DIANA tools. (**B**) Interaction between HOXC-AS1 and miR-99a-3p detected by the dual-luciferase reporter gene assay in AGS and SGC7901 cell lines. (**C**) MiR-99a-3p expression of in GES, AGS, SGC7901, SNU1 and MKN45 by qRT-PCR. (**D**) MiR-99a-3p expression in normal and tumorous gastric tissues from TCGA. (**E**) The correlation between HOXC-AS1 and miR-99a-3p. (**F**) MiR-99a-3p expression after downregulating HOXC-AS1 by qRT-PCR. ns represents no significance. * *p* < 0.05; ** *p* < 0.01; *** *p* < 0.001.

Considering that lncRNAs often function as sponges of microRNAs, the possible microRNAs that HOXC-AS1 may sponge were predicted by DIANA tools. We found a binding domain for miR-99a-3p (Figure 3A). In order to sustain the interaction between HOXC-AS1 and miR-99a-3p, a dual-luciferase reporter gene assay was implemented. As illustrated in Figure 3B, miR-99a-3p, when bound at a specific site in HOXC-AS1, led to significantly decreased luciferase activity, which was not distinctly influenced under the mutation of the binding site. As a result, we inspected the expression of miR-99a-3p in normal and tumorous gastric cells and discovered that miR-99a-3p was downregulated in AGS, SGC7901, SNU1 and MKN45 cell lines (Figure 3C). In accordance with our hypothesis, determined in the TCGA, miR-99a-3p expression was reduced in tumorous gastric tissues relative to normal gastric tissues (Figure 3D). Although there was no statistical significance, the expression of miR-99a-3p had a trend of negative correlation with the expression of HOXC-AS1 (Figure 3E). Additionally, the expression of miR-99a-3p was markedly elevated when HOXC-AS1 was suppressed in AGS and SGC7901 cells (Figure 3F). The above results confirmed that HOXC-AS1 may directly sponge miR-99a-3p in GC.

### 3.4. MiR-99a-3p Regulated MMP8 in GC

**Figure 4:**
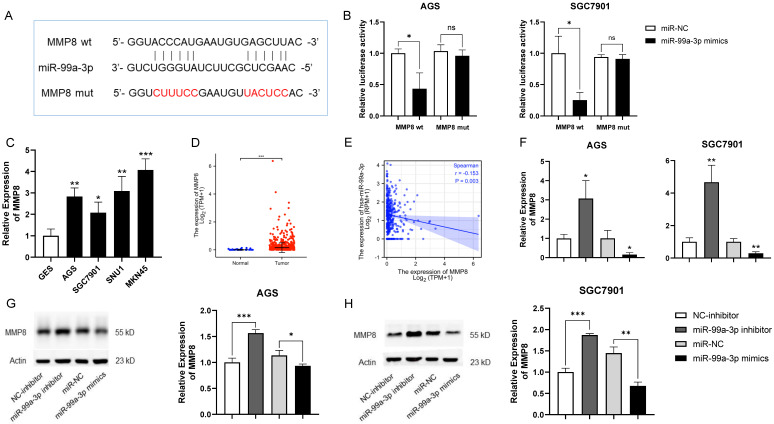
MiR-99a-3p regulated MMP8 in GC. (**A**) The forecast binding site between miR-99a-3p and MMP8 using TargetScan. (**B**) Interaction between miR-99a-3p and MMP8 explored by the dual-luciferase reporter gene assay in AGS and SGC7901 cell lines. (**C**) MMP8 expression in GES, AGS, SGC7901, SNU1 and MKN45 by qRT-PCR. (**D**) MMP8 expression in normal and tumorous gastric tissues from TCGA. (**E**) The relevance of miR-99a-3p and MMP8 expression. (**F**) MMP8 expression after overexpression and downregulation of miR-99a-3p by qRT-PCR. (**G**,**H**) MMP8 expression under overexpression and downregulation of miR-99a-3p in AGS and SGC7901 cell lines by western blot. Full blotting could be found at Figures S1–S3. ns represents no significance. * *p* < 0.05; ** *p* < 0.01; *** *p* < 0.001.

It was reported that microRNAs could restrain the translation or attract the degradation of mRNA, changing the expression of target genes, including oncogenic and tumor suppressive genes, which are closely connected with numerous malignancies [20]. Therefore, we used TargetScan to filter potential target genes regulated by miR-99a-3p, exposing that MMP8 contained a binding site for miR-99a-3p (Figure 4A). Later, the binding between miR-99a-3p and MMP8 was assessed by a dual-luciferase reporter assay. The overexpression of miR-99a-3p restrained the luciferase activity, while a similar effect was not observed when the predicted binding site for miR-99a-3p in MMP8 was mutated in AGS and SGC7901 cells (Figure 4B). As previously mentioned, MMP8 expression, as has been reported in cell experiments and described in the TCGA, was high in tumorous gastric cells and tissues compared to normal tissues and cells (Figure 4C,D). In addition, the expression of MMP8 was clearly negatively related to miR-99a-3p (Figure 4E). Afterwards, qRT-PCR and western blot were carried out to validate our conclusions. After suppression of miR-99a-3p with an inhibitor, the expression of MMP8 was evidently enhanced. Whereas, while miR-99a-3p was upregulated with mimics, the expression of MMP8 was congruously decreased (Figure 4F). A similar outcome was also achieved by western blot (Figure 4G,H). In general, MMP8 should be considered as a target gene of miR-99a-3p in GC.

### 3.5. HOXC-AS1 Promoted GC by Managing miR-99a-3p/MMP8 Axis

**Figure 5:**
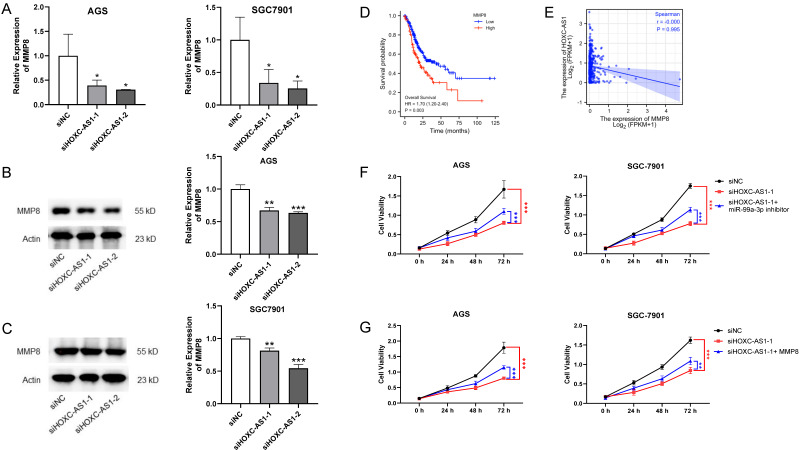
HOXC-AS1 promoted cell progression of GC by targeting the miR-99a-3p/MMP8 axis. (**A**) The expression of MMP8 following downregulation of HOXC-AS1 by qRT-PCR. (**B**,**C**) MMP8 expression following downregulation of HOXC-AS1 in AGS and SGC7901 cell lines by western blot. Full blotting could be found at Figures S4–S7. (**D**) Kaplan–Meier analysis for overall survival of GC patients based on the expression of MMP8. (**E**) The correlation of HOXC-AS1 and MMP8. (**F**,**G**) Rescue experiment of CCK-8 through co-transfecting miR-99a-3p inhibitor or overexpressing MMP8 in AGS and SGC7901. SiNC and siHOXC-AS1 were transfected as control. * *p* < 0.05; ** *p* < 0.01; *** *p* < 0.001.

After demonstration of the interaction among HOXC-AS1, miR-99a-3p and MMP8, we predicted them to be functioning as a ceRNA network. In consequence, we tried to probe the impact of the ceRNA network on GC progression. qRT-PCR and western blot disclosed that MMP8 was reduced at both the mRNA and protein level following knockdown of HOXC-AS1 (Figure 5A–C). Moreover, patients with highly expressed MMP8 were likely to have a shorter overall survival in the TCGA (Figure 5D). However, the expression of MMP8 did not show correlation with the expression of HOXC-AS1 (Figure 5E). Furthermore, when HOXC-AS1 was downregulated, proliferation of AGS and SGC7901 cells was also reduced; however, proliferation was subsequently rescued by transfecting miR-99a-3p inhibitor or upregulating MMP8 (Figure 5F,G). This study clearly confirmed that HOXC-AS1 is prominently involved in GC progression through targeting the miR-99a-3p/MMP8 axis.

## 4. Discussion

GC continues to be a major cancer burden worldwide and the large number of GC cases demand lifestyle interventions, such as smoking cessation and dietary changes [21]. A comprehensive therapy consisting of surgery, radiotherapy, chemotherapy and biologic therapy is suggested to be applied clinically to GC [22]. However, the 5-year survival rates of GC remain low as a result of the difficulty of an early diagnosis and the occurrence of early metastasis [23]. Therefore, novel therapies for GC treatment can have global implications.

According to prior research, lncRNAs take part in the occurrence and development of GC. In this study, we identified that HOXC-AS1 was overexpressed in GC. Currently, there are only a few reports about the underlying mechanism of HOXC-AS1 in the development of tumors. It has been reported to accelerate the advancement of nasopharyngeal carcinoma and prostate cancer [15,16]. Additionally, the expression of HOXC-AS1 was elevated by inhibition of EIF4AIII, thereby promoting cell proliferation and EMT in GC [17]. Coincidentally, suppression of HOXC-AS1 inhibited GC proliferation and metastasis both in vitro and in vivo [18]. Consistent with these studies, we found that with a poor prognosis, HOXC-AS1 was overexpressed in GC tissues from the TCGA. Moreover, we also found HOXC-AS1 to be upregulated in GC cell lines by qRT-PCR. In addition, we demonstrated by CCK-8 and colony formation assay that downregulation of HOXC-AS1 significantly suppressed GC cell proliferation.

Considering the function of lncRNAs as sponges for microRNAs, we discovered that HOXC-AS1 may directly sponge miR-99a-3p (Figure 3A,B). It was reported that miR-99a-3p loaded exosomes affected cell viability, metastasis and vascular formation ability of invasive pituitary adenoma (IPA) [24]. Studies also revealed that non-SMC condensin I complex subunit G (NCAPG) could be the downstream target of miR-99a-3p in prostate cancer cells, and distinctly influenced proliferation, migration and invasion [25]. MiR-99a-3p was also reported to be correlated with longer overall survival of colorectal cancer patients [26]. MiR-99a-3p also affected metastasis of papillary thyroid carcinoma by accommodating integrin subunit alpha 2 (ITGA2) expression and localization [27]. Nevertheless, except for the establishment of a microRNA-based signature for predicting the prognosis of GC, there has been little research regarding the specific effect of miR-99a-3p in GC. In our study, miR-99a-3p was found to be downregulated in gastric tissues and was strongly upregulated following knockdown of HOXC-AS1 (Figure 3C,D,F). Although the expression correlation between HOXC-AS1 and miR-99a-3p did not show significant association, a trend of negative correlation could be observed. One reason may be that the database was based on the expression from gastric tissues, while our investigation was conducted on cell lines.

As for the function of miR-99a-3p, MMP8 was affirmed to bind with miR-99a-3p in our research (Figure 4A,B). MMP8, called neutrophil collagenase or collagenase-2, encodes one member of the MMP protein family that unhinges triple helical type I collagen, other extracellular matrices (ECM) and plentiful non-ECM substrates [28]. These proteins are involved in embryonic development, reproduction, tissue remodeling and disease processes, such as arthritis [29]. In breast, skin and oral tongue cancer, substantial evidence reveals that MMP8 inhibits invasion and proliferation of cancer cells and reduces the probability of metastasis by eliminating non-structural substrates [30,31,32,33]. On the contrary, expression of MMP8 related positively to the expression of transforming growth factor β1 (TGF-β1) in hepatocellular carcinoma tissues, enhancement of which was related to cancer stage, metastasis and recurrence time [34]. With regard to GC, an excessively high or low molar ratio of serum MMP8 to tissue inhibitor of metalloproteinase-1 (TIMP-1) predicted a worse prognosis [35]. It was suggested that polymorphisms in MMP2, 3, and 8 could promote occurrence and fatality by affecting the activity of enzymes in GC [36]. The probable mechanism of MMP8 mediated promotion of GC may be through MMP8 driven upregulation of TGF-β1 by stimulation of the PI3K/Akt/Rac1 pathway, leading to EMT, and promotion of invasion and migration. MMP8 could also regulate Ephrin-B1, which transduces signals associated with cell adhesion and angiogenesis, to reveal some tumorigenic influences [37]. In addition, the intracellular C-terminus of ephrin-B1, which was necessary to cancer invasion, was reported to manage the MMP8 liberation from cells through activating the cell trafficking regulator Arf1 [38]. In our research, MMP8 was found overexpressed in gastric tissues and was clearly downregulated following upregulation of miR-99a-3p or knockdown of HOXC-AS1 (Figure 4C–H and Figure 5A–C). The correlation of expression between miR-99a-3p and MMP8 had valid statistical significance; however, no obvious correlation between HOXC-AS1 and MMP8 was found in the TCGA. The discordance between our research and the database may be due to the limited choices of database and samples. Besides, gastric cancer subtypes have been shown to have different mutational, clinical and epidemiological profiles. Subtyping may be relevant to the lack of correlation between HOXC-AS1 and MMP8 expression, as the reported regulation mechanism may be active only in a distinguished subset of samples. Functionally, we validated the HOXC-AS1/miR-99a-3p/MMP8 axis in our rescue experiment with the CCK-8 (Figure 5E,F). Since the expression of miR-99a-3p was reported to be regulated by diaporine A [19], it was reasonable to speculate that regulating miRNAs by natural agents may be a new strategy for cancer treatment. As for the feasible application of the outcomes, a nanocarrier system may be a potential method for delivery of the gene-targeted oligodeoxynucleotides in GC therapy [39].

## 5. Conclusions

Generally, a new ceRNA network consisting of lncRNA HOXC-AS1, microRNA miR-99a-3p and mRNA MMP8 was constituted. Overexpression of HOXC-AS1 would accordingly sponge greater quantities of miR-99a-3p, leading to the upregulation of MMP8, eventually facilitating the progress of GC.

## Data Availability

The datasets of gastric cancer for this study are available in TCGA (https://www.cancer.gov/about-nci/organization/ccg/research/structural-genomics/tcga, accessed on 23 June 2021).

## Supplementary Materials

The following supporting information can be downloaded at: https://www.mdpi.com/article/10.3390/cancers14143534/s1, Figure S1: MMP8 expression under overexpression and downregulation of miR-99a-3p in AGS cell line. Figure S2: MMP8 expression under overexpression and downregulation of miR-99a-3p in SGC7901 cell line. Figure S3: Actin expression under overexpression and downregulation of miR-99a-3p in AGS and SGC7901 cell lines. Figure S4: MMP8 expression following downregulation of HOXC-AS1 in AGS cell line. Figure S5: Actin expression following downregulation of HOXC-AS1 in AGS cell line. Figure S6: MMP8 expression following downregulation of HOXC-AS1 in SGC7901 cell line. Figure S7: Actin expression following downregulation of HOXC-AS1 in SGC7901 cell line.
