# Supplementary material for: LncRNA HOXC-AS1 Sponges miR-99a-3p and Upregulates MMP8, Ultimately Promoting Gastric Cancer
Source: Cancers (Basel). 2022 Jul 20;14(14):3534. doi: 10.3390/cancers14143534 (PMC9321533; doi:10.3390/cancers14143534)
Supplement: Supplementary file 1 [file cancers-14-03534-s001.zip › cancers-1738044-supplementary.pdf]

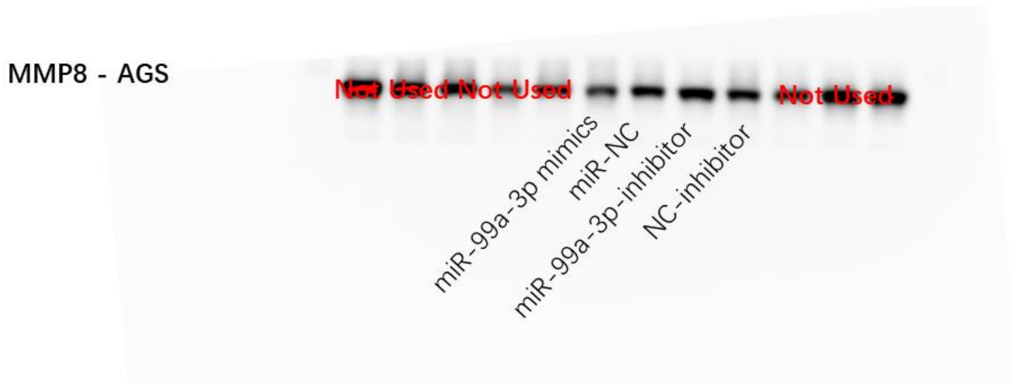

Figure S1. MMP8 expression under overexpression and downregulation of miR-99a-3p in AGS cell line.

MMP8 – SGC7901

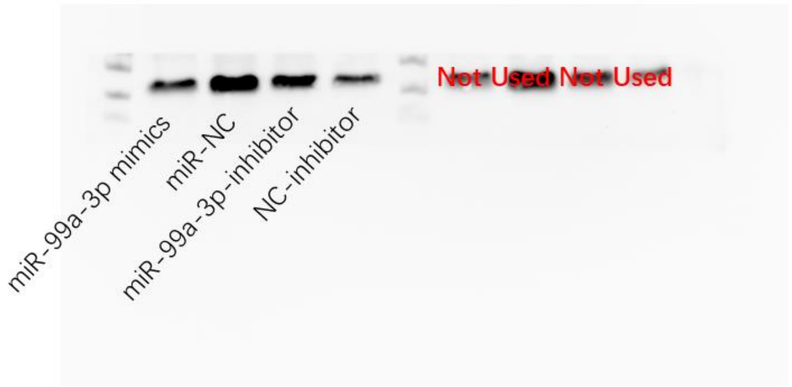

Figure S2. MMP8 expression under overexpression and downregulation of miR-99a-3p in SGC7901 cell line.

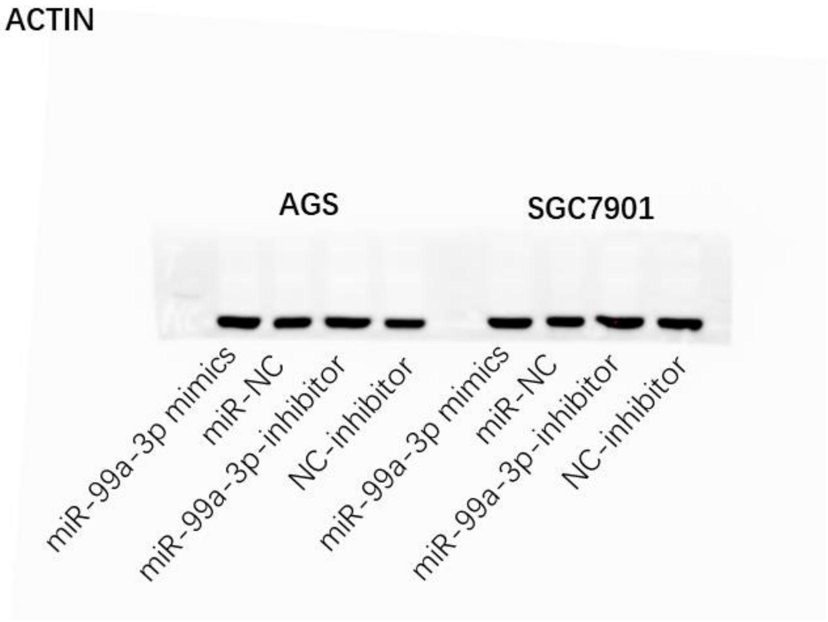

Figure S3. Actin expression under overexpression and downregulation of miR-99a-3p in AGS and SGC7901 cell lines.

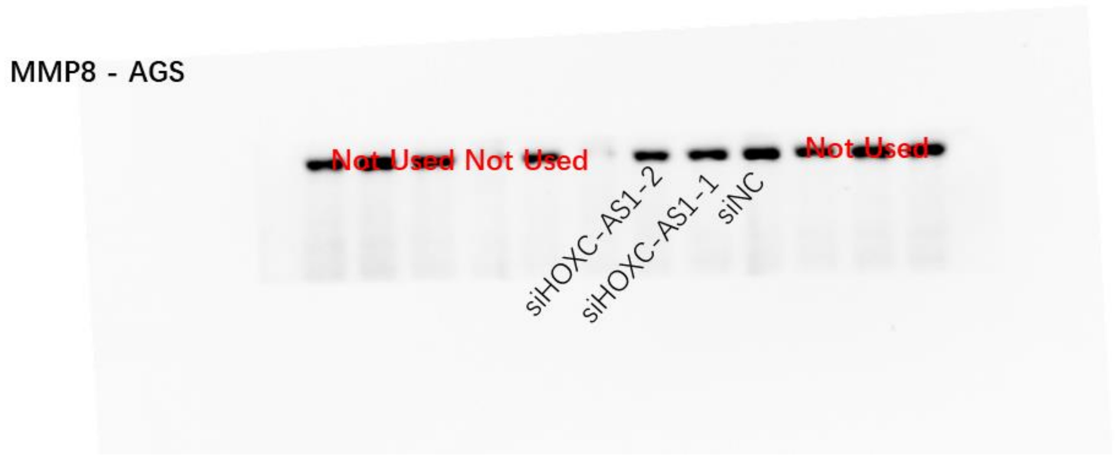

Figure S4. MMP8 expression following downregulation of HOXC-AS1 in AGS cell line.

ACTIN - AGS

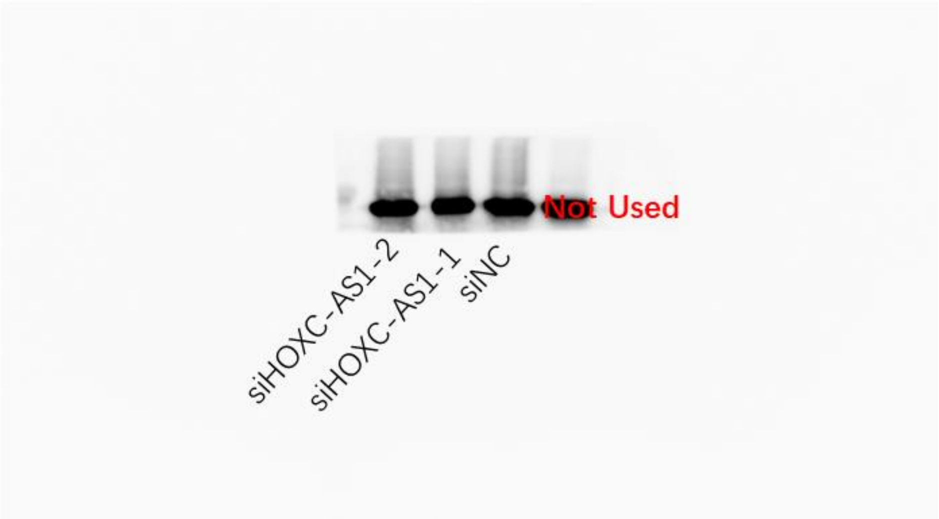

Figure S5. Actin expression following downregulation of HOXC-AS1 in AGS cell line.

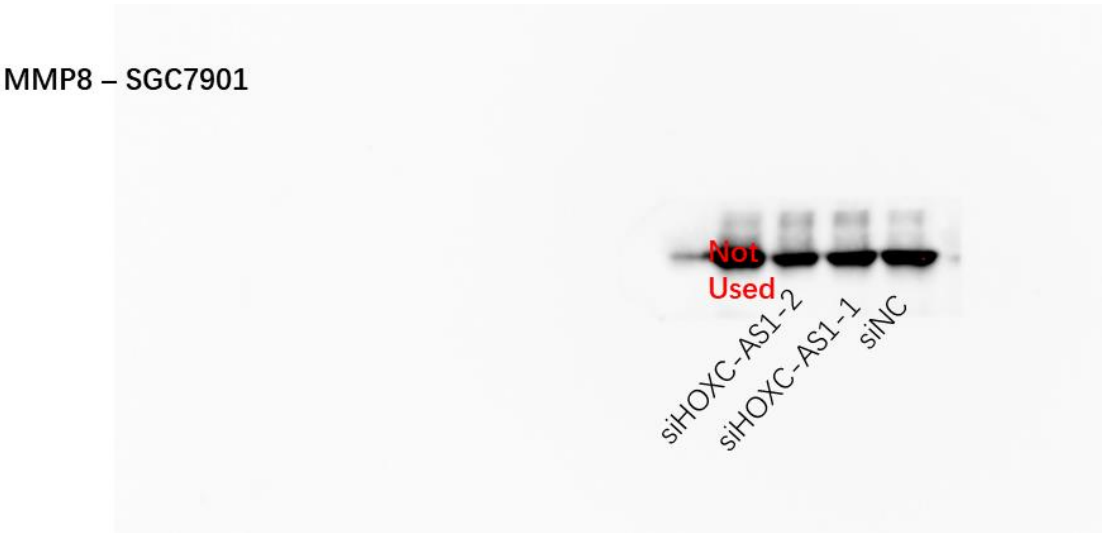

Figure S6. MMP8 expression following downregulation of HOXC-AS1 in SGC7901 cell line.

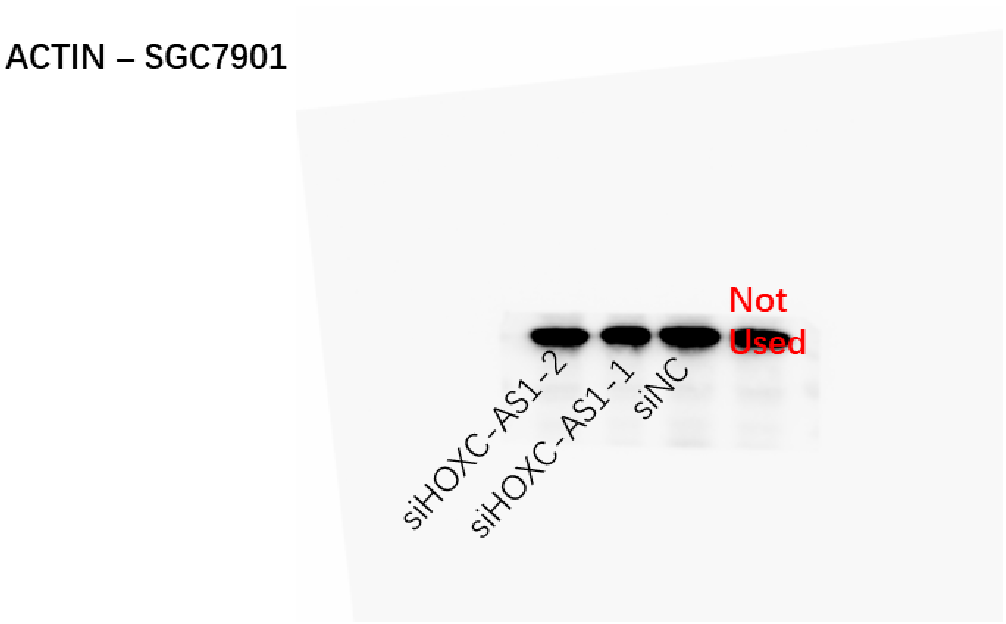

Figure S7. Actin expression following downregulation of HOXC-AS1 in SGC7901 cell line.
